# 1*H*-Pyrazol-2-ium hydrogen oxalate

**DOI:** 10.1107/S1600536812023136

**Published:** 2012-05-26

**Authors:** Chun-Hua Yu, Run-Qiang Zhu

**Affiliations:** a Ordered Matter Science Research Center, College of Chemistry and Chemical Engineering, Southeast University, Nanjing 211189, People’s Republic of China

## Abstract

In the title compound, C_3_H_5_N_2_^+^·C_2_HO_4_^−^, the anions form centrosymmetric dimers through cyclic O—H⋯O hydrogen-bonding associations [graph set *R*_2_^2^(10)]. These dimers are then linked through a cyclic *R*_4_^2^(10) N—H⋯O hydrogen-bonding association involving two cations and the carboxyl O-atom acceptors of separate anions, giving chain structures extending across the (111) plane.

## Related literature

For general background to ferroelectric organic frameworks, see: Fu *et al.* (2009 ▶); Ye *et al.* (2006 ▶); Zhang *et al.* (2008 ▶, 2010 ▶). For graph-set analysis, see: Etter *et al.* (1990 ▶).
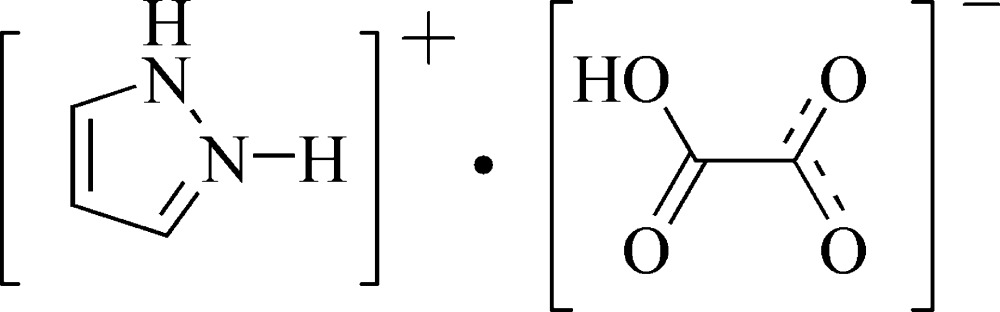


## Experimental

### 

#### Crystal data

C_3_H_5_N_2_^+^·C_2_HO_4_^−^
*M* *_r_* = 158.12
Triclinic, 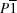

*a* = 3.7286 (7) Å
*b* = 9.836 (2) Å
*c* = 10.487 (2) Å
α = 117.35 (3)°
β = 97.01 (3)°
γ = 93.65 (3)°
*V* = 335.92 (14) Å^3^
*Z* = 2
Mo *K*α radiation
μ = 0.14 mm^−1^
*T* = 293 K
0.26 × 0.22 × 0.14 mm

#### Data collection

Rigaku SCXmini CCD diffractometer
Absorption correction: multi-scan (*CrystalClear*; Rigaku, 2005 ▶) *T*_min_ = 0.965, *T*_max_ = 0.993
3484 measured reflections
1527 independent reflections
702 reflections with *I* > 2σ(*I*)
*R*_int_ = 0.063

#### Refinement

*R*[*F* ^2^ > 2σ(*F* ^2^)] = 0.095
*wR*(*F* ^2^) = 0.285
*S* = 1.07
1527 reflections
101 parameters
H-atom parameters constrained
Δρ_max_ = 0.48 e Å^−3^
Δρ_min_ = −0.29 e Å^−3^

### 

Data collection: *CrystalClear* (Rigaku, 2005 ▶); cell refinement: *CrystalClear*; data reduction: *CrystalClear*; program(s) used to solve structure: *SHELXS97* (Sheldrick, 2008 ▶); program(s) used to refine structure: *SHELXL97* (Sheldrick, 2008 ▶); molecular graphics: *DIAMOND* (Brandenburg & Putz, 2005 ▶); software used to prepare material for publication: *SHELXL97*.

## Supplementary Material

Crystal structure: contains datablock(s) I, global. DOI: 10.1107/S1600536812023136/zs2209sup1.cif

Structure factors: contains datablock(s) I. DOI: 10.1107/S1600536812023136/zs2209Isup2.hkl

Supplementary material file. DOI: 10.1107/S1600536812023136/zs2209Isup3.cml

Additional supplementary materials:  crystallographic information; 3D view; checkCIF report

## Figures and Tables

**Table 1:** Hydrogen-bond geometry (Å, °)

*D*—H⋯*A*	*D*—H	H⋯*A*	*D*⋯*A*	*D*—H⋯*A*
N1—H1*A*⋯O1^i^	0.86	1.86	2.709 (5)	170
N2—H2*A*⋯O1^ii^	0.86	1.92	2.715 (5)	153
O4—H4⋯O3^iii^	0.82	1.95	2.679 (5)	147

Symmetry codes: (i) 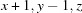; (ii) 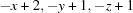; (iii) 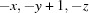.

**Fig. 1.**
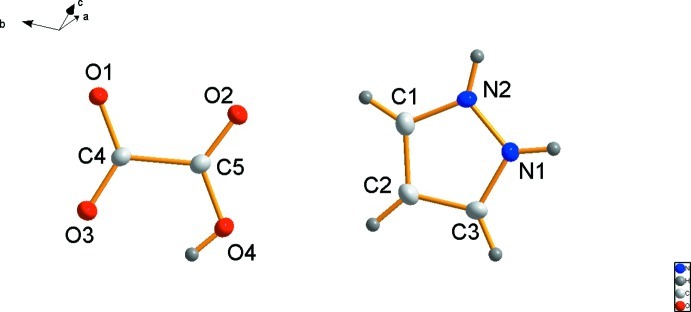
The molecular structure and atom-numbering scheme for the title compound, with the displacement ellipsoids drawn at the 30% probability level.

**Fig. 2.**
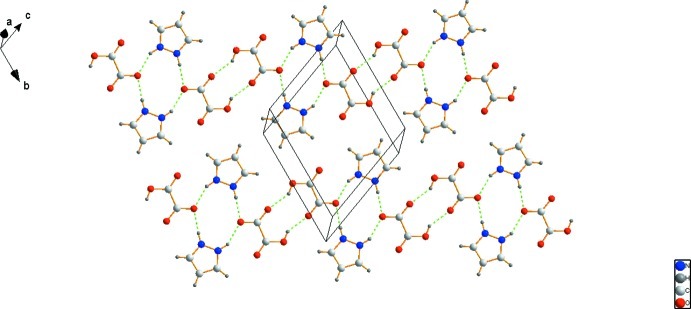
The packing of the title compound in the unit cell viewed down the a axis. Hydrogen bonds are shown as dashed lines.

## supplementary crystallographic information

### Comment

As a contribution to a search for new ferroelectric materials (Fu *et al.*, 2009; Ye *et al.*, 2006; Zhang *et al.*, 2008, 2010), we have synthesized the title salt, C_3_H_5_N_2_^+^. C_2_HO_4_^-^ from a 1:1 stoichiometric reaction of pyrazole with oxalic acid and the structure is reported here.

In the structure of the title compound (Fig.1) the molecules are organized in a one-dimensional chain structure involving both inter-anionic and cation–anion hydrogen-bonding associations (Table 1). The anions form centrosymmetric dimers through cyclic O—H···O hydrogen-bonding associations [graph set *R*^2^_2_(10) (Etter *et al.*, 1990)]. These dimers are then linked through a cyclic *R*^2^_4_(10) N—H···O hydrogen-bonding association involving two cations and the carboxyl O-atom acceptors of separate anions, giving one-dimensional chain structures extending across the (111) plane (Fig. 2).

### Experimental

A mixture of pyrazole (0.68 g, 10 mmol) and oxalic acid (0.95 g, 10 mmol) in water was stirred for several days at ambient temperature. Colourless crystal plates of the title compound suitable for X-ray analysis were obtained.

### Refinement

Hydrogen atom positions were calculated and allowed to ride on their parent atoms with aromatic C—H = 0.93 Å, N—H = 0.86 Å and O—H = 0.82 Å, and with *U*_iso_(H) = 1.2*U*_eq_(C or N) and *U*_iso_(H) = 1.5*U*_eq_(O).

### Crystal data

**Table:**

C_3_H_5_N_2_^+^·C_2_HO_4_^−^	*Z* = 2
*M**_r_* = 158.12	*F*(000) = 164
Triclinic, *P*1	*D*_x_ = 1.563 Mg m^−^^3^
Hall symbol: -P 1	Mo *K*α radiation, λ = 0.71073 Å
*a* = 3.7286 (7) Å	Cell parameters from 1527 reflections
*b* = 9.836 (2) Å	θ = 2.4–27.5°
*c* = 10.487 (2) Å	µ = 0.14 mm^−^^1^
α = 117.35 (3)°	*T* = 293 K
β = 97.01 (3)°	Sheet, colourless
γ = 93.65 (3)°	0.26 × 0.22 × 0.14 mm
*V* = 335.92 (14) Å^3^	

### Data collection

**Table:**

Rigaku SCXmini CCD diffractometer	1527 independent reflections
Radiation source: fine-focus sealed tube	702 reflections with *I* > 2σ(*I*)
Graphite monochromator	*R*_int_ = 0.063
CCD_Profile_fitting scans	θ_max_ = 27.5°, θ_min_ = 3.9°
Absorption correction: multi-scan (*CrystalClear*; Rigaku, 2005)	*h* = −4→4
*T*_min_ = 0.965, *T*_max_ = 0.993	*k* = −12→12
3484 measured reflections	*l* = −13→13

### Refinement

**Table:**

Refinement on *F*^2^	Primary atom site location: structure-invariant direct methods
Least-squares matrix: full	Secondary atom site location: difference Fourier map
*R*[*F*^2^ > 2σ(*F*^2^)] = 0.095	Hydrogen site location: inferred from neighbouring sites
*wR*(*F*^2^) = 0.285	H-atom parameters constrained
*S* = 1.07	*w* = 1/[σ^2^(*F*_o_^2^) + (0.1349*P*)^2^ + 0.0952*P*] where *P* = (*F*_o_^2^ + 2*F*_c_^2^)/3
1527 reflections	(Δ/σ)_max_ < 0.001
101 parameters	Δρ_max_ = 0.48 e Å^−^^3^
0 restraints	Δρ_min_ = −0.29 e Å^−^^3^

### Special details

**Table:**

Geometry. All e.s.d.'s (except the e.s.d. in the dihedral angle between two l.s. planes) are estimated using the full covariance matrix. The cell e.s.d.'s are taken into account individually in the estimation of e.s.d.'s in distances, angles and torsion angles; correlations between e.s.d.'s in cell parameters are only used when they are defined by crystal symmetry. An approximate (isotropic) treatment of cell e.s.d.'s is used for estimating e.s.d.'s involving l.s. planes.
Refinement. Refinement of *F*^2^ against ALL reflections. The weighted *R*-factor *wR* and goodness of fit *S* are based on *F*^2^, conventional *R*-factors *R* are based on *F*, with *F* set to zero for negative *F*^2^. The threshold expression of *F*^2^ > σ(*F*^2^) is used only for calculating *R*-factors(gt) *etc*. and is not relevant to the choice of reflections for refinement. *R*-factors based on *F*^2^ are statistically about twice as large as those based on *F*, and *R*- factors based on ALL data will be even larger.

### Fractional atomic coordinates and isotropic or equivalent isotropic displacement parameters (Å^2^)

**Table:**

	*x*	*y*	*z*	*U*_iso_*/*U*_eq_	
N1	1.0872 (11)	0.0136 (5)	0.3057 (4)	0.0416 (11)	
H1A	1.1595	−0.0585	0.3230	0.050*	
N2	1.1523 (11)	0.1643 (5)	0.3992 (4)	0.0412 (11)	
H2A	1.2729	0.2054	0.4865	0.049*	
C1	0.9992 (15)	0.2395 (6)	0.3352 (6)	0.0493 (15)	
H1	1.0047	0.3461	0.3773	0.059*	
C2	0.8307 (15)	0.1355 (7)	0.1967 (6)	0.0505 (15)	
H2	0.7020	0.1567	0.1277	0.061*	
C3	0.8931 (15)	−0.0076 (7)	0.1818 (6)	0.0477 (14)	
H3	0.8131	−0.1021	0.0993	0.057*	
O1	0.3997 (10)	0.8001 (4)	0.3600 (3)	0.0475 (10)	
O2	0.6353 (11)	0.5351 (4)	0.3418 (4)	0.0577 (12)	
O3	0.0807 (11)	0.6588 (4)	0.1355 (4)	0.0581 (11)	
O4	0.3270 (11)	0.4012 (4)	0.1179 (4)	0.0554 (11)	
H4	0.1905	0.4187	0.0612	0.083*	
C4	0.2907 (14)	0.6762 (6)	0.2446 (5)	0.0402 (13)	
C5	0.4374 (14)	0.5304 (6)	0.2400 (5)	0.0425 (13)	

### Atomic displacement parameters (Å^2^)

**Table:**

	*U*^11^	*U*^22^	*U*^33^	*U*^12^	*U*^13^	*U*^23^
N1	0.049 (3)	0.033 (2)	0.041 (2)	0.0045 (19)	−0.0029 (19)	0.019 (2)
N2	0.048 (3)	0.034 (2)	0.031 (2)	0.005 (2)	−0.0023 (19)	0.0087 (19)
C1	0.053 (3)	0.045 (3)	0.056 (4)	0.011 (3)	0.006 (3)	0.029 (3)
C2	0.053 (4)	0.052 (4)	0.048 (3)	0.010 (3)	−0.002 (3)	0.027 (3)
C3	0.047 (3)	0.042 (3)	0.041 (3)	0.004 (2)	−0.006 (2)	0.011 (3)
O1	0.062 (2)	0.033 (2)	0.037 (2)	0.0087 (17)	−0.0074 (17)	0.0110 (18)
O2	0.076 (3)	0.043 (2)	0.045 (2)	0.0122 (19)	−0.0127 (19)	0.018 (2)
O3	0.077 (3)	0.042 (2)	0.044 (2)	0.0123 (19)	−0.0126 (19)	0.0155 (19)
O4	0.075 (3)	0.032 (2)	0.045 (2)	0.0098 (19)	−0.0082 (19)	0.0113 (19)
C4	0.047 (3)	0.032 (3)	0.035 (3)	0.005 (2)	0.005 (2)	0.011 (2)
C5	0.050 (3)	0.033 (3)	0.039 (3)	0.003 (2)	0.004 (3)	0.013 (3)

### Geometric parameters (Å, º)

**Table:**

N1—C3	1.325 (6)	C2—H2	0.9300
N1—N2	1.333 (5)	C3—H3	0.9300
N1—H1A	0.8601	O1—C4	1.256 (6)
N2—C1	1.319 (6)	O2—C5	1.202 (6)
N2—H2A	0.8600	O3—C4	1.239 (6)
C1—C2	1.372 (7)	O4—C5	1.317 (6)
C1—H1	0.9300	O4—H4	0.8200
C2—C3	1.380 (8)	C4—C5	1.549 (7)
			
C3—N1—N2	109.3 (4)	C3—C2—H2	127.4
C3—N1—H1A	125.3	N1—C3—C2	108.0 (5)
N2—N1—H1A	125.4	N1—C3—H3	126.0
C1—N2—N1	108.4 (4)	C2—C3—H3	126.0
C1—N2—H2A	125.8	C5—O4—H4	109.5
N1—N2—H2A	125.8	O3—C4—O1	127.0 (5)
N2—C1—C2	109.1 (5)	O3—C4—C5	117.3 (4)
N2—C1—H1	125.4	O1—C4—C5	115.7 (4)
C2—C1—H1	125.4	O2—C5—O4	122.4 (5)
C1—C2—C3	105.2 (5)	O2—C5—C4	122.1 (5)
C1—C2—H2	127.4	O4—C5—C4	115.4 (4)
			
C3—N1—N2—C1	0.1 (6)	O3—C4—C5—O2	−178.2 (5)
N1—N2—C1—C2	0.0 (6)	O1—C4—C5—O2	1.9 (8)
N2—C1—C2—C3	0.0 (6)	O3—C4—C5—O4	1.0 (7)
N2—N1—C3—C2	−0.1 (6)	O1—C4—C5—O4	−178.9 (4)
C1—C2—C3—N1	0.1 (6)		

### Hydrogen-bond geometry (Å, º)

**Table:**

*D*—H···*A*	*D*—H	H···*A*	*D*···*A*	*D*—H···*A*
N1—H1*A*···O1^i^	0.86	1.86	2.709 (5)	170
N2—H2*A*···O1^ii^	0.86	1.92	2.715 (5)	153
O4—H4···O3^iii^	0.82	1.95	2.679 (5)	147

Symmetry codes: (i) *x*+1, *y*−1, *z*; (ii) −*x*+2, −*y*+1, −*z*+1; (iii) −*x*, −*y*+1, −*z*.
